# A Retrospective Observational Study Using Administrative Databases to Assess the Risk of Spontaneous Abortions Related to Environmental and Socioeconomic Conditions

**DOI:** 10.3390/life13091853

**Published:** 2023-09-01

**Authors:** Massimo Giotta, Nicola Bartolomeo, Paolo Trerotoli

**Affiliations:** 1 School of Medical Statistics and Biometry, Department of Interdisciplinary Medicine, University of Bari Aldo Moro, 70124 Bari, Italy; 2 Department of Precision and Regenerative Medicine, University of Bari Aldo Moro, 70124 Bari, Italy; 3 Department of Interdisciplinary Medicine, University of Bari Aldo Moro, 70124 Bari, Italy; nicola.bartolomeo@uniba.it (N.B.); paolo.trerotoli@uniba.it (P.T.)

**Keywords:** spontaneous abortion, reproductive health, miscarriage risk factors, adverse pregnancy outcomes, administrative databases, exposure, air pollution, environmental risk, deprivation index, socioeconomic inequalities

## Abstract

Miscarriage is one of the most frequent adverse events that occurs during pregnancy. This retrospective study aimed to verify if the environmental and socioeconomic conditions related to geographical areas where women live, and the socio-demographic and clinical factors play a role in the risk of spontaneous abortion (SA). The analyses were conducted by hospital discharge records (HDRs) from public and private hospitals in Apulia from 1 January 2021 to 31 December 2021. Women with an age over 40 years old had a major risk of SA compared with women under 18 years (OR 2.30, IC95%1.16–4.54). A reduction in the risk of SA was found for women with an endocrinological or metabolic disease (OR 0.28, 95% CI 0.19–0.41), while genetic disease greatly increases the risk (OR 9.63, IC95% 1.98–46.86). The greatest risk of spontaneous abortion was found in the province of Taranto compared to the province of Foggia (OR 2.01, 95% CI 1.52–2.64). The provinces with a higher risk of SA in the multiple comparisons were Taranto, Brindisi, and BAT. Municipalities with socioeconomic disadvantages classified as very low, low, and medium had a higher risk of SA compared to the municipalities with a high disadvantage. In conclusion, our study indicates the possible association between SA rate and environmental conditions. Additionally, the socioeconomic, clinical, and demographic factors were related to the risk of SAs.

## 1. Introduction

Miscarriage is one of the most frequent adverse events that occur during pregnancy. “Miscarriage” is attached to a considerable number of different and legally mandated definitions [1]. Two terms are used to describe the scenario of fetal death: miscarriage and stillbirth. While miscarriage refers to early pregnancy losses, stillbirth refers to fetal deaths that occur later on during pregnancy [2,3]. These discrepancies impact the outcome of accurate data collection in vital registration systems, stillbirth registries, surveys, and research studies [4]. The World Health Organization (WHO) established a definition for “miscarriage” as follows: “The expulsion from the mother of an embryo or fetus weighing 500 g or less, indicating a gestational age of up to 20 completed weeks, without any signs of life” [5]. In Italy, a spontaneous abortion refers to a pregnancy that has ended before the 180th full day of amenorrhea [6]. Legal provisions that regulate the management of spontaneous abortions within the healthcare system exist in Italy and ensure support for and assistance of women experiencing this painful experience. It is important to underline that, in the legislation in force in Italy, the difference between an “abortion” and “stillbirth” is placed at 25 + 5 weeks of gestational age; a product of conception without vitality expelled up to 180 days of gestational age is considered an abortion by law. From the 181st day, it is considered stillborn [6].

Fetal death is principally caused by a genetic disorder [7]. However, socioeconomic status, as an indicator of wealth, education, and employment, is associated with an individual’s health, and its impact on pregnancy outcomes has also been observed in the literature, as people with high socioeconomic deprivation levels are more likely to exhibit risky behavioral patterns and have limited access to health services [8]. Work also has an impact on pregnancy, especially if a job is stressful or physically or psychologically demanding. In this case, an increased risk of miscarriage, preterm delivery, low birth weight, and pre-eclampsia outcomes are observed [9]. Environmental factors are additional potential risk factors for the occurrence of miscarriages. Exposure to fine particulate (PM2.5) and other pollutants has been associated with an increase in the risk of miscarriages in the literature [10]. A systematic review that analyzed 35 human studies suggested that, on the one hand, exposure to carbon monoxide (CO), PM10, and PM2.5 may be associated with a higher risk of spontaneous abortion; on the other hand, NO_2_ and SO_2_ exposures presented conflicting evidence [11]. Furthermore, the effect of pollutants may have an influence on specific subpopulations versus others. A study conducted in the United States [12] showed that NO2 exposure had a major effect on non-Whites, individuals living below the poverty line, and individuals with a lower level of education.

In Puglia, some municipalities exist where pollution plays an important role in health outcomes. In 2011, the Ministry of Health established the “Sentieri Project” where experienced epidemiologists evaluated the effect of pollutants on mortality rates. The cities under analysis in this project were Barletta, Bari, Brindisi, Manfredonia, and Taranto. Many researchers have studied how environmental pollution in the cities of Taranto and Brindisi had an impact on the state of health of the general population. The main consequences analyzed in the research were hospital admissions rates for acute or chronic diseases [13,14] and the rate of mortality for chronic disease [15,16,17]. The study focused on heart or lung failure [18] and cancer [19,20,21,22] cases.

The first aim of this study is to verify if the environmental conditions related to geographical areas where women live play a role, along with the socio-demographic factors, in the risk of miscarriages, using the available administrative data. The secondary objective is to evaluate if socioeconomic deprivation also has an impact on spontaneous abortion outcomes.

## 2. Materials and Methods

We performed a retrospective observational study using the relevant data extracted from the administrative healthcare databases of the Regional Epidemiological Observatory. The data were gathered and stored within the Regional Information System, with access regulated by a regional policy to enable their utilization for the epidemiological research of inpatients from public and private hospitals in Puglia from 1 January 2021 to 31 December 2021. Only hospital discharge records (HDRs) for spontaneous abortion (SA) and delivery were included in this study, and we identified women with main or secondary diagnoses of spontaneous (ICD9CM 634.XX) or missed (ICD9-CM 632) abortions as spontaneous abortion cases, while childbirth was the main or secondary diagnosis of normal delivery (ICD9-CM 650) or multiple gestation (ICD9-CM651.XX) cases. Voluntary abortion was classified by ICD9-CM 635-636.

We calculated the rate of SAs for municipalities as the ratio between the number of SAs in a municipality and the total number of those conceived in that specific municipality (SA plus voluntary abortion and childbirth).

To evaluate the effect of chronic diseases on the risk of SA outcomes, we linked the HDRs with the database of patients who benefited from an exemption from payment of health services for disease during the year under analysis in the study. The deterministic record linkage was used to link the two databases using the patient’s univocal code as the key field. The univocal code was assigned for every patient after the anonymization of the record for the protection of privacy. The list of illnesses and criteria to apply for this ticket exemption was defined by the Ministry of Health [23]. We categorized all of the exemptions on basis of the diseases with which they correlated: circulatory (codes: 0A02, 0B02, 0C02, 0A31, 0031, 036), cardiac (code: 021), psychiatric (codes: 005, 014, 044), neurologic (codes: 011, 017, 029, 046, 038), rheumatologic (codes: 006, 028, 030, 054, 067, 060), respiratory (codes: 007, 024, 057), endocrine–metabolic (codes: 025, 026 027, 032, 035, 012, 013, 022, 039, 056, 001), kidney failure (codes: 023, 061), HIV (code: 020), neoplastic (code: 048), disability (codes: 049, 051), gynecologic (code: 063), genetic (codes: 062, 065, 066, 018, 008, RDG), hepatic–pancreatic (codes: 042, 016), and autoimmune (codes: 009, 045, 059, 041, 003).

In order to assess the relevant socioeconomic impacts, we employed the index developed by Caranci et al. [24] and revised in 2011 by Rosano et al. [25]. This deprivation index (DI) considered five factors, such as education, home ownership, population density, unemployment rate, and the prevalence of single-parent families. The DI was computed at the level of the census, taking into account the data obtained from the 2011 census. The DI could be used to describe typical social characteristics; however, its main use in the study was as a proxy to assess the level of individual social disadvantages, especially in areas where data at the individual level were difficult to access or were unavailable. Under this assumption, we calculated the municipal DI as a weighted average of the DIs of the census sections, using the resident populations in each census section as weights [26]. Subsequently, the DI was categorized into four classes using quartiles of the regional distribution: high (H), representing DIs lower than −1.331; medium (M), encompassing DIs ranging from −1.331 to −0.78; low (L), including DIs between −0.78 and −0.202; and very low (VL), comprising DIs higher than −0.202.

Categorical data were presented as frequency and percentage, and for the comparisons between groups, chi-squared or Fisher’s exact tests were utilized, as appropriate. To explore the risk of SA, the univariate and multivariable generalized linear models (GLM) were employed. The GLM model, originally formulated by Nelder and Wedderburn [27], uses a binomial distribution with a logit link function. The primary covariates included in the model were age (categorized into six groups: <18, 18–24, 25–29, 30–34, 35–39, and >40 years old), class of the deprivation index, educational level, province in which they live, marital status (maiden, married, separate, divorced, and widow), and chronic diseases (endocrinological, neoplastic, genetic, immune, neurodegenerative, respiratory, cardiocirculatory, liver, psychiatric, rheumatological, endometriosis). The significant variables in the univariate analysis were entered into the multivariable model. The results of the GLM model were shown as odds ratios and their 95% confidence interval (CI) for each variable. Pairwise multiple comparisons were adjusted according to the Tukey correction. A *p*-value < 0.05 was considered statistically significant. The data management, descriptive statistics, and regression modeling processes were conducted using SAS/STAT version 9.4 for PC (SAS Institute, Cary, NC, USA).

## 3. Results

The number of HDRs registered during 2021 was 391,563, but only 25,961 were included in this study for the inclusion criteria. The median age of the whole study sample was 33 years [IQR 29–36]. In total, 92.8% of HDRs are related to childbirth and the median age for this woman was 32 years [IQR 29–36], instead, 7.2% had a spontaneous abortion with a median age of 35 years [IQR 30–39]. Only 19 (0.001%) women used a technique of assisted reproduction. The main characteristics of the patients under analysis are shown in Table 1.

The main characteristics are different between women who had an SA and women who had a child (all *p*-value < 0.001). An increase in the percentage of abortion compared to childbirth was observed in the province of Taranto (20.0% vs. 13.8%), Lecce (23.0% vs. 18.4%), Brindisi (10.6% vs. 9.3%), and Barletta–Andria–Trani (BAT) (11.4% vs. 10.8%). In the other province, instead was observed a percentage of childbirth major than SA: Bari (32.0% vs. 22.7%) and Foggia (15.7% vs. 12.3%).

Compared the chronic disease between the woman who had an abortion compared to a woman who had not, a difference was observed only for endocrinologic–metabolic disease (*p* < 0.0001) and genetic disease (*p* = 0.01) (Table 2).

In the group of the women who had a childbirth, the percentages of the child being born alive were the 99.6% while the 0.03% born dead (Table 3).

Univariate and multivariable GLMs were applied to estimate the probability of SA in relation to the province of residence, class of socioeconomic deprivation and other possible covariates such as age class, level of education, marital status, and anamnesis for all possible chronic diseases. All significant variables at univariate analysis resulted independently related with the probability of SA by multivariable model (Table 4).

**Figure 1:**
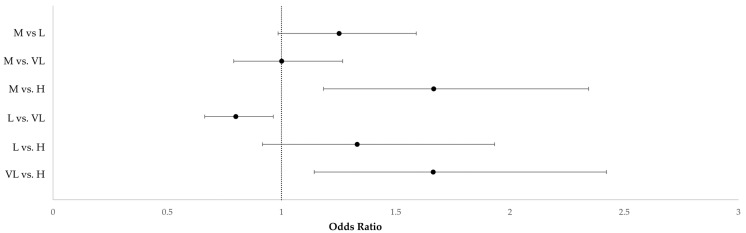
Forest plot of the adjusted odds ratios and their 95% CI for the comparison between the class of Deprivation Index (DI). H (high), DIs lower than −1.331; M (medium), DIs from −1.331 to −0.78; L (low), DIs from −0.78 to −0.202; VL (very low), DIs higher than −0.202.

Municipalities with socioeconomic disadvantages classified as very low, low, and medium had a higher risk of SA compared to the municipalities with a high disadvantage (VL: OR 1.67, IC95% 1.25–2.16; L: OR 1.33, IC95% 1–1.77; M: OR 1.66, IC95% 1.28–2.16). All classes of DI comparison are reported in Figure 1.

**Figure 2:**
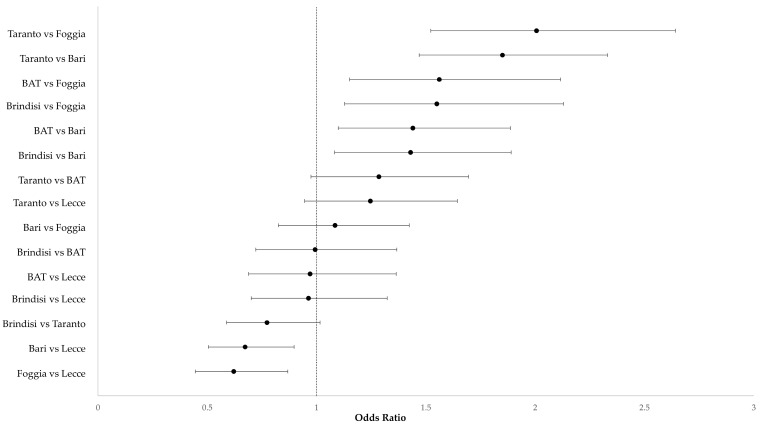
Forest plot of the adjusted odds ratios and their 95% CI for the comparison between the provinces of Apulia. BAT, Barletta–Andria–Trani.

In addition, women with an age over 40 years old had a major risk of SA compared with women under 18 years (OR 2.30, IC95%1.16–4.54). Instead a reduction in SA was observed for young women (18–24 years vs. <18 years: OR 0.43, IC95% 0.21–0.86). A reduction in the risk of SA was found for women with an endocrinological or metabolic disease (OR 0.28, 95% CI 0.19–0.41), while genetic disease greatly increased the risk (OR 9.63, IC95% 1.98–46.86). The province of Bari has a lower risk of SA than the other provinces (Brindisi vs. Bari: OR 1.43, 95% CI 1.08–1.89; Taranto vs. Bari: OR 1.85, 95% CI 1.47–2.33; BAT vs. Bari: OR 1.44, 95% CI 1.10–1.89). The greatest risk of spontaneous abortion was found in the province of Taranto compared to the province of Foggia (OR 2.01, 95% CI 1.52–2.64). The provinces with a higher risk of SA in the multiple comparison were Taranto, Brindisi, and BAT. All province comparisons are reported in Figure 2.

A reduction in the risk of spontaneous abortion was found in married women compared to separated women (OR 0.52, 95% CI 0.29–0.41) and unmarried woman compared to separated women (OR 0.46, 95% CI 0.26–0.81). This last one is statistically significant only in the univariate logistic model. All odds ratio with their CI95% of the pairwise multiple comparison from univariate and multivariable models are shown in Table S1.

**Figure 3:**
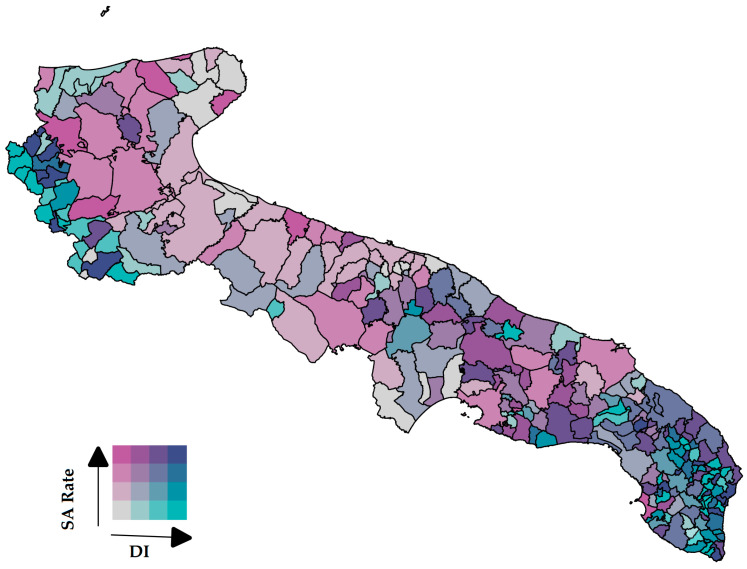
Bivariate choropleth map of Apulia. Relationship between deprivation index and SA rate in 2021. SA, spontaneous abortion; DI, deprivation index.

To evaluate the geographic association between the level of deprivation and the incidence of SA throughout the region, we drew a bivariate choropleth map (Figure 3). 

The north (except the north-west) and the center of Puglia are characterized by lower socio-economic hardship and a medium-low SA rate. In the south-eastern area of the region, there is instead the greater presence of municipalities characterized by a higher level of deprivation and a higher incidence of SA. The already known areas at environmental risk, mainly Taranto and Brindisi, appeared in the map with a higher level of SA rated, even with a low level of deprivation.

## 4. Discussion

We conducted a study to identify the factors that were related to the risk of SAs, and to evaluate the relationship between childbirth and socio-economic and environmental conditions related to geographical areas.

There were mainly two causes of SAs: problems related to the fetus and problems related to the mother. In our study, we did not analyze fetal abnormalities; however, it was crucial to remember that the leading cause of SAs for fetal malformations was associated with chromosomal abnormalities [28]. The mother’s genome was also important in this context because women with a history of genetic abnormalities present a higher risk of experiencing SAs than women without these diseases. Some researchers hypothesized that chromosomic anomalies could provoke recurrent SAs and could be associated with a family history of abortions [29]. Some studies [30,31] have pointed out that the inherited tendency for recurrent miscarriage due to thrombophilia involves genetic mutations in blood coagulation factors II and V, as well as natural anticoagulants such as antithrombin, protein C, and protein S. A major risk of miscarriage was also found for another type of hypercoagulant disease: the antiphospholipid syndrome [32,33]. In general, Ghazi et al. [34] showed that prenatal exposure to pollutants was associated with epigenetic alterations, oxidative stress in the placenta, aging, and alterations in energy metabolism levels. Additionally, congenital anomalies may be associated with mothers being exposed to air pollution. In the previous decade, numerous researchers investigated the association between an increase in congenital anomalies and air pollution levels [35]; in fact, several systemic reviews have been published on this issue [36,37,38,39]. A recent systematic review and meta-analysis [40] showed the effects of pollutants, mainly concerning orofacial defects, cardiac deformities (e.g., tetralogy of Fallot, ventricular septal defects, pulmonary valve stenosis), and limb defects. Various studies explored how the mother’s disease impacted SA outcomes, especially in the endocrinological field. In a historical review edited in 1987 [41], the author explained the link between diabetes and a higher incidence of abortions. When a woman with diabetes desires to become pregnant, she should schedule periodic glucometabolic checks from the early period of pregnancy. For this reason, the risk of SAs for women with metabolic syndrome is reduced.

Age plays a role in spontaneous abortion results because chromosomal abnormalities are related to age [42]. Women older than 40 years old are a major risk group in comparison to younger women, regardless of their reproductive history [43]. The likelihood of an SA is highly influenced by the age of the mother and exhibits a significant recurrence pattern. Furthermore, the risk of an SA tends to increase after certain adverse pregnancy outcomes. The underlying causes shared between Sas and other pregnancy complications can stem from biological conditions or unmeasured common risk factors [44].

Other socio-demographic parameters (e.g., education level, socioeconomic status) were evaluated in accordance with the results obtained by the other authors. It has been widely confirmed that a high-level education reduces the risk of Sas, because people with a high-level education have better access to health services and can better understand how to deal with the situation [45]. In accordance with the results of the other authors [45] who studied the risk of Sas among Italian and immigrant women, we believe that education has an indirect impact on the health of both the mother and fetus. Furthermore, in a Danish study [46], women studied for a period shorter than 10 years had a major risk of a spontaneous abortion than women we studied for over 10 years.

Further studies underlined the effects of socioeconomic deprivation on the risk of Sas [47,48,49]. In a study conducted in Manitoba, Canada [50], a reduction in Sas was observed for women with a higher-level socioeconomic status. There is a possibility that women with a higher-level socioeconomic status are more likely to identify very early pregnancies, leading to the detection and timely care of early pregnancy losses.

In our study, we observed that the risk of Sas was higher in the less socioeconomically disadvantaged class. We hypothesized that this effect might be associated with job outcomes because employed women may have less socioeconomic disadvantages. In fact, studies conducted during that time increasingly underlined an increase in the risk of abortion or childbirth outcomes among working women [51,52]. This risk was higher in women who worked in the textile sector. Furthermore, the ergonomic factor [53], cold working environments, and performing physically demanding tasks [54] can contribute to abortions and stillbirths [55,56].

The working environment was not the only factor that required careful monitoring to offer a safe place for pregnant women. It has been amply demonstrated in the research that various environmental factors (physical and chemical) influence pregnancy outcomes, which requires a particular balance of endocrine and immunological functions [45]. In fact, some substances, such as benzene and polycyclic aromatic hydrocarbons, are endocrine-disrupting chemicals (EDCs) [57]. The growth of the fetus is sensitive to pollutants so that, in women exposed to particular environmental pollutants, an increase in preterm births, a decrease in birth weight, and the determination of congenital anomalies were observed [11]. In a prospective cohort study, it was discovered that there was a noteworthy increase in the hazard ratio of 1.13 for each interquartile range increase at a PM2.5 level, leading to a greater risk of spontaneous abortions during the entire pregnancy [58]. Two retrospective cohort studies also revealed significant increases in the adjusted odds ratios (AORs) for spontaneous abortions among women exposed to PM10 levels higher than 56.72 μg/m^3^, compared to those exposed to PM10 levels equal to or below 56.72 μg/m^3^. The AORs were reported as 5.05 and 2.59 in the respective studies [59,60]. Furthermore, the impact of pollutants was also observed on the weight at birth. In cities with a high number of industrial plants, babies were at a major risk of being born with a low birth weight (<2500 g), in comparison to those living in other cities [61]. In our study, we observed an increase in the risk of AS for the provinces of Taranto and Brindisi, both classified as Sites of National Interest (SIN) based on the characteristics of the sites, the levels and severity of the pollutants present, the impact on the surrounding environment in terms of health and ecological risks, as well as damage to cultural and environmental heritage [62]. However, the ongoing research in this area highlights the dynamic nature of the results, which may vary between studies. Additionally, individual susceptibility to environmental exposure can vary due to certain factors, such as genetics, lifestyle, and duration of exposure [11].

It is important to recognize the limitations present in our study. Our reliance on administrative data, susceptible to the accuracy of disease coding, was one of these limitations. As a result, data quality can be affected by the skill and experience of practitioners, potentially introducing bias into the reported case counts and rates (under- or over-reporting). Furthermore, it is important to note that this study exclusively included women who sought care at the hospital. Other women may have opted for outpatient care through their obstetric or primary care providers. Spontaneous pregnancy loss occurring in the early weeks of gestation might remain undocumented if a woman is unaware of the pregnancy and perceives the event as a regular menstrual cycle. These factors inevitably restricted the total number of cases documented during our study. Furthermore, the weakness of the health databases is that they do not contain information that could be really valuable for investigating the role of specific elements of the metabolic profile of women who suffer a miscarriage.

## 5. Conclusions

Our study indicates the possible association between spontaneous abortion cases and environmental conditions related to geographical areas. Further research is needed to better understand the relationship between air pollution exposure and pregnancy loss rates, and to establish better health policies. Additionally, the socioeconomic and demographic factors played a role in determining the risk of SAs. Women who lived in municipalities with a low DI had a high-level risk of spontaneous abortions, while a reduction in the risk of an SA was observed in women with a history of endocrinologic disorders or even with a low level of education. Future interventions should specifically target women residing in critical areas with additional risk factors for SAs.

## Figures and Tables

**Table 1:** Main characteristics according to the principal diagnosis. Comparison between SA and childbirth.

Parameter	Spontaneous Abortion (N = 1876)	Born (N = 24,085)	*p*-Value
**DI Class**			
<−1.33	88 (4.69)	1356 (5.63)	<0.0001
−1.33 to −0.78	382 (20.36)	3751 (15.57)	
−0.78 to −0.20	377 (20.1)	5148 (21.37)	
>−0.20	1029 (54.85)	13,830 (57.45)	
**Age**			
<18	11 (0.59)	92 (0.38)	<0.0001
18–24	114 (6.08)	2361 (9.8)	
25–29	265 (14.13)	4735 (19.66)	
30–34	486 (25.91)	8511 (35.34)	
35–39	614 (32.73)	6360 (26.41)	
>40	386 (20.58)	2026 (8.41)	
**Province of residence**			
Brindisi	198 (10.55)	2242 (9.31)	<0.0001
Taranto	376 (20.04)	3394 (13.84)	
BAT	216 (11.35)	2602 (10.8)	
Bari	426 (22.7)	7710 (32.01)	
Foggia	231 (12.31)	3775 (15.67)	
Lecce	432 (23.03)	4422 (18.36)	
**Educational level**			
Whitout degree	10 (0.58)	52 (0.25)	0.0003
Primary	31 (1.79)	250 (1.19)	
Secondary	486 (28.11)	5427 (25.85)	
High school	726 (41.99)	9810 (46.73)	
Graduate	49 (2.83)	577 (2.75)	
High graduation degree	427 (24.7)	4877 (23.23)	
**Marital status**			
Unmarried	752 (43.44)	8630 (38.95)	<0.0001
Married	930 (53.73)	13,213 (59.64)	
Legaly separated	29 (1.68)	154 (0.7)	
Divorced	17 (0.98)	146 (0.66)	
Widow	3 (0.17)	13 (0.06)	

Data are shown as n(%). BAT, Barletta–Andria–Trani.

**Table 2:** Frequency of principal chronic illness found in women in our sample. Comparison between SA and childbirth.

Chronic Disease	Spontaneous Abortion (N = 1876)	Born (N = 24,085)	*p*-Value
Endocrinological diseases	30(1.6)	1208(5.02)	<0.0001
Neoplastic diseases	4(0.21)	34(0.14)	0.3515
Genetic diseases	3(0.16)	4(0.02)	0.0106
Immune diseases	2(0.11)	14(0.06)	0.3235
Neurodegenerative diseases	2(0.11)	9(0.04)	0.1863
Respiratory diseases	2(0.11)	10(0.04)	0.2135
Cardiocircolatory diseases	3(0.16)	16(0.07)	0.1541
Liver diseases	0(0)	7(0.03)	1
Psychiatric diseases	0(0)	2(0.01)	1
Rheumatological diseases	0(0)	1(0)	1
Endometriosis	0(0)	1(0)	1

Data are shown as n(%).

**Table 3:** Frequency of the childbirth by delivery outcome.

Delivery Outcome	Code	N	%
Liveborn	v27.0	22,121	98.4
Stillborn	v27.1	80	0.4
Twins: both liveborn	v27.2	283	1.3
Twins: one liveborn, one stillborn	v27.3	4	0.02
Twins: both stillborn	v27.4	1	0.01
Multiple birth: all liveborn	v27.5	4	0.02
Multiple birth: some liveborn	v27.6	0	0
Multiple birth: all stillborn	v27.7	0	0
Multiple birth: unspecified	v27.9	2	0.01

**Table 4:** Type III effect of the univariate and multivariable general linear model applied to the probability of SA.

Parameters	Univariate		Multivariable	
	Chi-Square	*p*-Value	Chi-Square	*p*-Value
Class of deprivation	29.48	<0.001	25.07	<0.001
Class of age	344.83	<0.001	338.56	<0.001
Educational level	19.04	<0.001	16.28	0.001
Marital status	37.6	0.001	35.94	<0.001
Province	130.65	<0.001	82.93	<0.001
Endocrinologic disease	39.9652	<0.001	62.13	<0.001
Genetic disease	8.7963	0.003	6.34	0.011
Neoplastic diseases	0.6179	0.4318	-	-
Immune diseases	0.6443	0.4222	-	-
Neurodegenerative diseases	1.8077	0.1788	-	-
Respiratory diseases	1.487	0.2227	-	-
cardiocircolatory diseases	1.9547	0.1621	-	-
Liver diseases	0.005	0.9438	-	-
Psychiatric diseases	0.0031	0.9559	-	-
Rheumatological diseases	0.0032	0.9549	-	-
Endometriosis	0.0032	0.9549	-	-

## Data Availability

Data sharing is not applicable to this article.

## Supplementary Materials

The following supporting information can be downloaded at: https://www.mdpi.com/article/10.3390/life13091853/s1, Table S1: Results of the univariate and multivariable general linear models applied to estimate the probability of spontaneous abortion.

## References

[1] Barfield W.D., Committee on Fetus and Newborn (2011). Standard Terminology for Fetal, Infant, and Perinatal Deaths. Pediatrics.

[2] American College of Obstetricians and Gynecologists (2009). ACOG Practice Bulletin No. 102: Management of Stillbirth. Obs. Gynecol..

[3] Graafmans W.C., Richardus J.H., Macfarlane A., Rebagliato M., Blondel B., Verloove-Vanhorick S.P., Mackenbach J.P., EuroNatal Working Group (2001). Comparability of Published Perinatal Mortality Rates in Western Europe: The Quantitative Impact of Differences in Gestational Age and Birthweight Criteria. BJOG.

[4] International Infant Mortality Rates: Bias from Reporting Differences—PMC. https://www.ncbi.nlm.nih.gov/pmc/articles/PMC1615029/.

[5] Kumar G. (2011). Early Pregnancy Issues for the MRCOG and Beyond.

[6] Stillbirth: Clinical Audit and Improvement of Care Practice. https://www.salute.gov.it/imgs/C_17_pubblicazioni_1390_annex.pdf.

[7] Griebel C.P., Halvorsen J., Golemon T.B., Day A.A. (2005). Management of Spontaneous Abortion. Am. Fam. Physician.

[8] Baba S., Noda H., Nakayama M., Waguri M., Mitsuda N., Iso H. (2011). Risk Factors of Early Spontaneous Abortions among Japanese: A Matched Case-Control Study. Hum. Reprod..

[9] Katz V.L. (2012). Work and Work-Related Stress in Pregnancy. Clin. Obs. Gynecol..

[10] Liang W., Liang H., Ou L., Chen B., Chen A., Li C., Li Y., Guan W., Sang L., Lu J. (2020). Development and Validation of a Clinical Risk Score to Predict the Occurrence of Critical Illness in Hospitalized Patients With COVID-19. JAMA Intern. Med..

[11] Grippo A., Zhang J., Chu L., Guo Y., Qiao L., Zhang J., Myneni A.A., Mu L. (2018). Air Pollution Exposure during Pregnancy and Spontaneous Abortion and Stillbirth. Rev. Environ. Health.

[12] Clark L.P., Millet D.B., Marshall J.D. (2014). National Patterns in Environmental Injustice and Inequality: Outdoor NO2 Air Pollution in the United States. PLoS ONE.

[13] Forastiere F., Faustini A. (2009). Gruppo collaborativo EpiAir [Short-term effects of air pollution on human health: From epidemiological research to epidemiological surveillance]. Epidemiol. Prev..

[14] Serinelli M., Gianicolo E.A.L., Cervino M., Mangia C., Portaluri M., Vigotti M.A. (2010). Acute effects of air pollution in Brindisi (Italy): A case-crossover analysis. Epidemiol. Prev..

[15] Gennaro V., Cervellera S., Cusatelli C., Miani A., Pesce F., De Gennaro G., Distante A., Vimercati L., Gesualdo L., Piscitelli P. (2022). Use of Official Municipal Demographics for the Estimation of Mortality in Cities Suffering from Heavy Environmental Pollution: Results of the First Study on All the Neighborhoods of Taranto from 2011 to 2020. Environ. Res.

[16] Galise I., Serinelli M., Morabito A., Pastore T., Tanzarella A., Laghezza V., Nocioni A., Giua R., Bauleo L., Bruno V. (2019). The Integrated Environmental Health Impact of emissions from a steel plant in Taranto and from a power plant in Brindisi, (Apulia Region, Southern Italy). Epidemiol. Prev..

[17] Leogrande S., Alessandrini E.R., Stafoggia M., Morabito A., Nocioni A., Ancona C., Bisceglia L., Mataloni F., Giua R., Mincuzzi A. (2019). Industrial Air Pollution and Mortality in the Taranto Area, Southern Italy: A Difference-in-Differences Approach. Environ. Int..

[18] Gianicolo E.A.L., Bruni A., Mangia C., Cervino M., Vigotti M.A. (2013). Acute Effects of Urban and Industrial Pollution in a Government-Designated “Environmental Risk Area”: The Case of Brindisi, Italy. Int. J. Environ. Health Res..

[19] Marinaccio A., Belli S., Binazzi A., Scarselli A., Massari S., Bruni A., Conversano M., Crosignani P., Minerba A., Zona A. (2011). Residential Proximity to Industrial Sites in the Area of Taranto (Southern Italy). A Case-Control Cancer Incidence Study. Ann. Ist. Super. Sanita.

[20] Martinelli D., Mincuzzi A., Minerba S., Tafuri S., Conversano M., Caputi G., Lopalco P.L., Quarto M., Germinario C., Prato R. (2009). Malignant Cancer Mortality in Province of Taranto (Italy). Geographic Analysis in an Area of High Environmental Risk. J. Prev. Med. Hyg..

[21] Pirastu R., Comba P., Iavarone I., Zona A., Conti S., Minelli G., Manno V., Mincuzzi A., Minerba S., Forastiere F. (2013). Environment and Health in Contaminated Sites: The Case of Taranto, Italy. J. Environ. Public Health.

[22] Giua R., Spartera M., Viviano G., Ziemacki G., Carbotti G. (2005). Cancer risk for coke-oven workers in the Taranto steel plant. Epidemiol. Prev..

[23] Definition and Update of the Essential Levels of Assistance, Referred to in Article 1, Paragraph 7, of Legislative Decree 30 December 1992, n. 502. https://www.gazzettaufficiale.it/eli/id/2017/03/18/17A02015/sg.

[24] Caranci N., Biggeri A., Grisotto L., Pacelli B., Spadea T., Costa G. (2010). The Italian deprivation index at census block level: Definition, description and association with general mortality. Epidemiol. Prev..

[25] Rosano A., Pacelli B., Zengarini N., Costa G., Cislaghi C., Caranci N. (2020). Update and review of the 2011 Italian deprivation index calculated at the census section level. Epidemiol. Prev..

[26] Bartolomeo N., Giotta M., Tafuri S., Trerotoli P. (2022). Impact of Socioeconomic Deprivation on the Local Spread of COVID-19 Cases Mediated by the Effect of Seasons and Restrictive Public Health Measures: A Retrospective Observational Study in Apulia Region, Italy. Int. J. Environ. Res. Public Health.

[27] Nelder J.A., Wedderburn R.W.M. (1972). Generalized Linear Models. J. R. Stat. Society. Ser. A (Gen.).

[28] Hao J., Zhang F., Chen D., Liu Y., Liao L., Shen C., Liu T., Liao J., Ma L. (2019). Association between Ambient Air Pollution Exposure and Infants Small for Gestational Age in Huangshi, China: A Cross-Sectional Study. Environ. Sci. Pollut. Res. Int..

[29] Woolner A.M.F., Nagdeve P., Raja E.-A., Bhattacharya S., Bhattacharya S. (2020). Family History and Risk of Miscarriage: A Systematic Review and Meta-Analysis of Observational Studies. Acta Obs. Gynecol. Scand..

[30] Liu X., Chen Y., Ye C., Xing D., Wu R., Li F., Chen L., Wang T. (2021). Hereditary Thrombophilia and Recurrent Pregnancy Loss: A Systematic Review and Meta-Analysis. Hum. Reprod..

[31] Lino F.L., Traina É., Barreto J.A., Moron A.F., Mattar R. (2015). Thrombophilic Mutations and Polymorphisms, Alone or in Combination, and Recurrent Spontaneous Abortion. Clin. Appl. Thromb. Hemost..

[32] Martinez-Zamora M.A., Peralta S., Creus M., Tassies D., Reverter J.C., Espinosa G., Cervera R., Carmona F., Balasch J. (2012). Risk of Thromboembolic Events after Recurrent Spontaneous Abortion in Antiphospholipid Syndrome: A Case-Control Study. Ann. Rheum. Dis..

[33] Di Prima F.A.F., Valenti O., Hyseni E., Giorgio E., Faraci M., Renda E., De Domenico R., Monte S. (2011). Antiphospholipid Syndrome during Pregnancy: The State of the Art. J. Prenat. Med..

[34] Ghazi T., Naidoo P., Naidoo R.N., Chuturgoon A.A. (2021). Prenatal Air Pollution Exposure and Placental DNA Methylation Changes: Implications on Fetal Development and Future Disease Susceptibility. Cells.

[35] Chen E.K.-C., Zmirou-Navier D., Padilla C., Deguen S. (2014). Effects of Air Pollution on the Risk of Congenital Anomalies: A Systematic Review and Meta-Analysis. Int. J. Environ. Res. Public. Health.

[36] Padula A.M., Tager I.B., Carmichael S.L., Hammond S.K., Lurmann F., Shaw G.M. (2013). The Association of Ambient Air Pollution and Traffic Exposures with Selected Congenital Anomalies in the San Joaquin Valley of California. Am. J. Epidemiol..

[37] Padula A.M., Tager I.B., Carmichael S.L., Hammond S.K., Yang W., Lurmann F., Shaw G.M. (2013). Ambient Air Pollution and Traffic Exposures and Congenital Heart Defects in the San Joaquin Valley of California. Paediatr. Perinat. Epidemiol..

[38] Gianicolo E.A.L., Mangia C., Cervino M., Bruni A., Andreassi M.G., Latini G. (2014). Congenital Anomalies among Live Births in a High Environmental Risk Area—A Case-Control Study in Brindisi (Southern Italy). Environ. Res..

[39] Schembari A., Nieuwenhuijsen M.J., Salvador J., de Nazelle A., Cirach M., Dadvand P., Beelen R., Hoek G., Basagaña X., Vrijheid M. (2014). Traffic-Related Air Pollution and Congenital Anomalies in Barcelona. Environ. Health Perspect..

[40] Ravindra K., Chanana N., Mor S. (2021). Exposure to Air Pollutants and Risk of Congenital Anomalies: A Systematic Review and Metaanalysis. Sci. Total Environ..

[41] Kalter H. (1987). Diabetes and Spontaneous Abortion: A Historical Review. Am. J. Obs. Gynecol..

[42] Pellestor F., Andréo B., Arnal F., Humeau C., Demaille J. (2003). Maternal Aging and Chromosomal Abnormalities: New Data Drawn from in Vitro Unfertilized Human Oocytes. Hum. Genet..

[43] Nybo Andersen A.M., Wohlfahrt J., Christens P., Olsen J., Melbye M. (2000). Maternal Age and Fetal Loss: Population Based Register Linkage Study. BMJ.

[44] Magnus M.C., Wilcox A.J., Morken N.-H., Weinberg C.R., Håberg S.E. (2019). Role of Maternal Age and Pregnancy History in Risk of Miscarriage: Prospective Register Based Study. BMJ.

[45] Caserta D., Ralli E., Matteucci E., Bordi G., Soave I., Marci R., Moscarini F. (2015). The Influence of Socio-Demographic Factors on Miscarriage Incidence among Italian and Immigrant Women: A Critical Analysis from Italy. J. Immigr. Minor. Health.

[46] Yang L., Tao T., Zhao X., Tao H., Su J., Shen Y., Tang Y., Qian F., Xiao J. (2020). Association between Fetal Chromosomal Abnormalities and the Frequency of Spontaneous Abortions. Exp. Ther. Med..

[47] Thomson K., Moffat M., Arisa O., Jesurasa A., Richmond C., Odeniyi A., Bambra C., Rankin J., Brown H., Bishop J. (2021). Socioeconomic Inequalities and Adverse Pregnancy Outcomes in the UK and Republic of Ireland: A Systematic Review and Meta-Analysis. BMJ Open.

[48] Smith L.K., Budd J.L.S., Field D.J., Draper E.S. (2011). Socioeconomic Inequalities in Outcome of Pregnancy and Neonatal Mortality Associated with Congenital Anomalies: Population Based Study. BMJ.

[49] Zheng D., Li C., Wu T., Tang K. (2017). Factors Associated with Spontaneous Abortion: A Cross-Sectional Study of Chinese Populations. Reprod. Health.

[50] Strumpf E., Lang A., Austin N., Derksen S.A., Bolton J.M., Brownell M.D., Chateau D., Gregory P., Heaman M.I. (2021). Prevalence and Clinical, Social, and Health Care Predictors of Miscarriage. BMC Pregnancy Childbirth.

[51] Ramji S. (1989). Socio-Economic and Environmental Determinants of Perinatal and Neonatal Mortality in India. Indian. Pediatr..

[52] Banerjee B., Dey T.K., Chatterjee P. (2005). Work Related Physical Exertion and Spontaneous Abortion. Indian. J. Public. Health.

[53] Goulet L., Thériault G. (1987). Association between Spontaneous Abortion and Ergonomic Factors. A Literature Review of the Epidemiologic Evidence. Scand. J. Work. Environ. Health.

[54] Working Conditions and Adverse Pregnancy Outcome: A Meta-Analysis—PubMed. https://pubmed.ncbi.nlm.nih.gov/10725502/.

[55] McDonald A.D., McDonald J.C., Armstrong B., Cherry N., Delorme C., D-Nolin A., Robert D. (1987). Occupation and Pregnancy Outcome. Br. J. Ind. Med..

[56] Seidel H. (1993). Selected Health Risks Caused by Long-Term, Whole-Body Vibration. Am. J. Ind. Med..

[57] Mantovani A., Stazi A.V., Macrì C., Maranghi F., Ricciardi C. (1999). Problems in Testing and Risk Assessment of Endocrine Disrupting Chemicals with Regard to Developmental Toxicology. Chemosphere.

[58] Ha S., Sundaram R., Buck Louis G.M., Nobles C., Seeni I., Sherman S., Mendola P. (2018). Ambient Air Pollution and the Risk of Pregnancy Loss: A Prospective Cohort Study. Fertil. Steril..

[59] Perin P.M., Maluf M., Czeresnia C.E., Nicolosi Foltran Januário D.A., Nascimento Saldiva P.H. (2010). Effects of Exposure to High Levels of Particulate Air Pollution during the Follicular Phase of the Conception Cycle on Pregnancy Outcome in Couples Undergoing in Vitro Fertilization and Embryo Transfer. Fertil. Steril..

[60] Perin P.M., Maluf M., Czeresnia C.E., Januário D.A.N.F., Saldiva P.H.N. (2010). Impact of Short-Term Preconceptional Exposure to Particulate Air Pollution on Treatment Outcome in Couples Undergoing in Vitro Fertilization and Embryo Transfer (IVF/ET). J. Assist. Reprod. Genet..

[61] Trerotoli P., Bartolomeo N., Leogrande S., Triggiani S., Mincuzzi A., Serio G., Minerba A.S. (2021). Survey of Low Birthweight and Extremely Low Birthweight Events in a High Environmental Risk Area of Apulia, Italy. Int. J. Environ. Res..

[62] Legislative Decree 3 April 2006, n. 152 Environmental Standards. https://www.gazzettaufficiale.it/dettaglio/codici/materiaAmbientale.

