# Supplementary material for: A Retrospective Observational Study Using Administrative Databases to Assess the Risk of Spontaneous Abortions Related to Environmental and Socioeconomic Conditions
Source: Life (Basel). 2023 Sep 1;13(9):1853. doi: 10.3390/life13091853 (PMC10532634; doi:10.3390/life13091853)
Supplement: Supplementary file 1 [file life-13-01853-s001.zip › life-2566296-supplementary.pdf]

Supplementary material

Table S1. Results of the univariate and multivariable General Linear Models applied to estimate the probability of Spontaneous Abortion.

Parameter	Comparison	UNIVARIATE		MULTIVARIABLE	
		p-value	OR[IC95%][1]	p-value	OR[IC95%][1]
**DI Class**	M vs.. L		0.64[0.47-0.87]		1.25[0.99-1.59]
	M vs. VL		0.89[0.65-1.21]		1[0.79-1.27]
	M vs. H	<0.0001	0.87[0.65-1.17]	<0.0001	1.67[1.18-2.34]
	L vs. VL		1.39[1.15-1.69]		0.8[0.66-0.96]
	L vs. H		1.37[1.17-1.61]		1.33[0.92-1.93]
	VL vs. H		0.98[0.84-1.16]		1.66[1.14-2.42]
**Age**	<18 vs. 18-24		2.48[0.96-6.4]		2.33[0.85-6.37]
	<18 vs. 25-29		2.14[0.85-5.4]		1.73[0.64-4.63]
	<18 vs. 30-34		2.09[0.84-5.25]		1.5[0.56-4]
	<18 vs. 35-39		1.24[0.5-3.1]		0.88[0.33-2.34]
	<18 vs. >40		0.63[0.25-1.58]		0.44[0.16-1.17]
	18-24 vs. 25-29		0.86[0.62-1.2]		0.74[0.52-1.06]
	18-24 vs. 30-34		0.85[0.62-1.15]		0.64[0.46-0.9]
	18-24 vs. 35-39	<0.0001	0.5[0.37-0.67]	<0.0001	0.38[0.27-0.53]
	18-24 vs. >40		0.25[0.19-0.35]		0.19[0.13-0.27]
	25-29 vs. 30-34		0.98[0.78-1.23]		0.87[0.68-1.11]
	25-29 vs. 35-39		0.58[0.47-0.72]		0.51[0.4-0.65]
	25-29 vs. >40		0.29[0.23-0.37]		0.25[0.19-0.33]
	30-34 vs. 35-39		0.59[0.49-0.71]		0.59[0.48-0.71]
	30-34 vs. >40		0.3[0.24-0.37]		0.29[0.23-0.36]
	35-39 vs. >40		0.51[0.42-0.62]		0.5[0.4-0.61]
**Province of residence**	BR vs. TA		0.78[0.6-1.02]		0.77[0.59-1.02]
	BR vs. BAT		1.08[0.81-1.45]		0.99[0.72-1.37]
	BR vs. BA		1.6[1.24-2.06]		1.43[1.08-1.89]
	BR vs. FG		1.44[1.08-1.92]		1.55[1.13-2.13]
	BR vs. LE		0.9[0.7-1.17]		0.96[0.7-1.32]
	TA vs. BAT		1.38[1.07-1.78]		1.29[0.97-1.7]
	TA vs. BA		2.04[1.65-2.52]		1.85[1.47-2.33]
	TA vs. FG	<0.0001	1.84[1.44-2.36]	<0.0001	2.01[1.52-2.64]
	TA vs. LE		1.15[0.93-1.43]		1.25[0.95-1.64]
	BAT vs. BA		1.48[1.16-1.9]		1.44[1.1-1.89]
	BAT vs. FG		1.34[1.01-1.77]		1.56[1.15-2.12]
	BAT vs. LE		0.84[0.65-1.08]		0.97[0.69-1.36]
	BA vs. FG		0.9[0.71-1.15]		1.08[0.83-1.42]
	BA vs. LE		0.57[0.46-0.69]		0.67[0.51-0.9]
	FG vs. LE		0.63[0.49-0.8]		0.62[0.45-0.87]
**Educational level**	low vs. medium		1.23[1.06-1.44]		1.26[1.07-1.48]
	low vs. high		1.04[0.88-1.24]		1.21[1-1.45]
	low vs. without	0.0003	0.47[0.19-1.16]	0.001	0.5[0.17-1.48]
	medium vs high		0.85[0.72-0.99]		0.96[0.81-1.13]
	medium vs. without		0.39[0.16-0.94]		0.4[0.13-1.18]
	high vs. without		0.45[0.19-1.11]		0.41[0.14-1.23]
**Marital status**	unmarried vs. married		1.24[1.08-1.42]		1.36[1.17-1.59]
	unmarried vs. legaly separeted		0.46[0.26-0.81]		0.71[0.39-1.29]
	unmarried vs. divorced		0.75[0.37-1.52]		1.18[0.57-2.44]
	unmarried vs. widow	<0.0001	0.38[0.07-2.17]	<0.0001	0.75[0.12-4.64]
	married vs. legaly separeted		0.37[0.21-0.65]		0.52[0.29-0.94]
	married vs. divorced		0.6[0.3-1.22]		0.86[0.42-1.79]
	married vs. widow		0.31[0.05-1.76]		0.55[0.09-3.4]
	legaly separeted vs. divorced		1.62[0.66-3.94]		1.66[0.66-4.16]

	legaly separeted vs.widow		0.82[0.13-5.1]		1.05[0.16-7.08]
	divorced vs. widow		0.51[0.08-3.31]		0.63[0.09-4.49]
**Endocrinologic disease**	Yes vs. No	<0.0001	0.31[0.21-0.44]	<0.0001	0.28[0.19-0.41]
**Genetic disease**	Yes vs. No	0.003	9.64[2.16-43.12]	0.0118	9.63[1.98-46.86]

[1]adjusted for multiple comparison with Tukey-Kramer

H (High), DIs lower than -1.331; M (Medium), DIs from -1.331 to -0.78; L (Low), DIs from -0.78 to -0.202; VL (Very Low), DIs higher than -0.202.
